# Influence of Baking Temperature and Formulation on Physical, Sensorial, and Morphological Properties of *Pogácsa* Cake: An Image Analysis Study

**DOI:** 10.3390/foods11030321

**Published:** 2022-01-24

**Authors:** Hanieh Amani, László Baranyai, Katalin Badak-Kerti, Amin Mousavi Khaneghah

**Affiliations:** 1 Department of Grains and Industrial Plants Processing, Hungarian University of Agriculture and Life Sciences, 1118 Budapest, Hungary; badakne.kerti.katalin@uni-mate.hu; 2 Department of Measurements and Process Control, Hungarian University of Agriculture and Life Sciences, 1118 Budapest, Hungary; baranyai.laszlo@uni-mate.hu; 3 Department of Food Science and Nutrition, Faculty of Food Engineering, University of Campinas, Campinas 13083-862, SP, Brazil

**Keywords:** image analysis, textural properties, pore volumetric ratio, porosity

## Abstract

*Pogácsa* is a high-demand bakery product with a unique texture, where crumb structure is a determining factor for its textural quality and consumer acceptability. At present, there is no non-destructive in-line inspection method for textural quality assessment of *pogácsa*. Therefore, this study was aimed to evaluate the texture of *pogácsa* using the image processing technique, which was prepared using different cheeses with varying moisture contents (MC) and was baked at 200 and 215 °C. Samples were assessed for textural, visual, physical, and sensorial properties. The findings indicated that the highest porosity (72.75%) was found for the sample baked at 215 °C with low-moisture cheese (58%), while the lowest porosity (32.66%) was observed for cheese-free sample baked at 200 °C. Pore volumetric ratio and MC showed strong correlations (*p* < 0.01) with hardness (−0.90 and −0.89), resilience (0.87 and 0.83), cohesiveness (0.84 and 0.82), springiness (0.87 and 0.90), gumminess (−0.92 and −0.92), and chewiness (−0.92 and −0.92), respectively. The pore volumetric ratio showed a strong correlation (*p* < 0.01) with reference porosity (0.71). Overall, the current study indicated that adding cheese with varying MC and baking temperature could affect the texture of *pogácsa* cake, which could be detected by image analysis.

## 1. Introduction

Over the past century, leavened bakery products are essential to families’ food baskets. This category encompasses products such as buns, cakes, and bread. The main ingredients used for their formulation are flour, biological or chemical leavening agents, and water. Some other ingredients such as egg, sugar, and shortening can also be added depending on the type and desired characteristics of the final product [1]. According to the Bakery and Confectionery Global Market Report, the worldwide market of bakery products was around 887.82 billion dollars in 2020. *Pogácsa* is one leavened traditional Hungarian cake, typically made of wheat flour, margarine, yeast, salt, and other ingredients. There is six percent salt in the recipe of *pogácsa* cake (based on the flour weight). Therefore, it has a salty taste. In addition, *pogácsa* is inelastic, slightly dense, with a particular texture, crispy surface, and tender core, giving characteristic and desired sensorial perception. Although this cake is regarded as a high-demand bakery product owing to its distinctive textural property, the studies focusing on the quality improvement and assessment of its physical and sensorial attributes are very limited [2].

As consumer awareness and expectation of high-quality bakery products increase, the importance of using accurate, non-destructive, and rapid quality assessment methods has become a big challenge for bakery manufactures [3,4]. The ingredients and baking conditions, particularly time and temperature, are the principal processing parameters, which significantly influence the quality attributes of the end product [5]. The most crucial quality attributes are color, shape, size, and crumb texture. Among these, crumb texture is the critical parameter for their quality assessment [1,6]. As discussed above, textural parameter, in particular, crumb texture, plays a vital role in the overall acceptance of *pogácsa*.

On the other hand, the changes in crumb pore structure may alter the consumer’s acceptability of *pogácsa*. Therefore, evaluating the relationship between crumb pore structure and the overall acceptability of the *pogácsa* becomes an important aspect of quality assessment and product formulation. Thus far, the quality assessment parameters such as crumb porosity and pore size distribution have been examined to understand the internal structure of the bakery products using different methodologies (e.g., human sensory evaluation, instrumental analysis). Each method, regardless of principle, has its pros and cons. For instance, the instrumental and sensorial approaches are usually time-consuming, expensive, and/or destructive, which may not suit in-line inspection [7,8]. Several relatively new techniques and methods such as hyperspectral imaging, ultrasonic technology, and spectroscopy have been successfully developed and utilized for quality inspection of bakery products; however, most of these methods are expensive and need advanced instruments with trained personnel [9]. Furthermore, the heterogeneity in the distribution of pores and variation in their size and structure can significantly affect the accuracy of these methods [1,10]. Therefore, given the above, there is a need for a convenient alternative method, which is non-destructive and also has higher accuracy and result consistency, suitable for in-line inspection in the industrial sector.

The computerized image analysis techniques are the core of the machine vision system, which can evaluate various features (e.g., color, shape, size, and pattern) from a digitalized picture [8,11]. This recent approach has the advantage of being non-destructive, rapid, and cheaper compared to the analytical methods. This method has successfully been applied in various bakery products to evaluate the morphological and textural qualities, grading, and identification in bakery products [12,13]. Studies have exhibited the potential of image processing in microstructural measurements of bakery products and its connection with the mechanical properties and sensorial attributes [9,14,15]. In our previous study, the effect of the baking parameters on *pogácsa* texture was evaluated using gray level co-occurrence matrix (GLCM) imaging analysis. The relationship between the instrumental texture parameters and GLCM was also assessed. The findings revealed that baking time and temperature change could significantly influence the product texture and change textural parameters. Therefore, the GLCM-based technique could be used as a non-destructive method for quality assessment of *pogácsa* [2]. Jha et al. [16] examined the effects of the proofing on the internal properties of the dough and baked bread via an image processing method. Authors found that with increasing proofing time, the dough porosity increased, which led to the increase in the specific volume and porosity of baked bread [16]. Esteller et al. [3] proposed an image analysis method to assess the impact of kefir addition on the porous properties of white bread. The physical, mechanical, and imaging evaluation results indicated that the increase in proofing time, the cell mean perimeter, cell mean area, light reflectance, chewiness, hardness, and specific volume changed [3]. In another study, Rahimi et al. [17] introduced an image analysis technique, which successfully evaluated the porosity characteristics of the leavened baked product prepared under different formulations and process conditions [17]. Ghaitaranpour et al. [18] studied the impact of air frying and deep-fat frying on doughnuts’ porous structure and porosity using the image analysis. The study revealed that the frying method could significantly affect the doughnut crumb pore characteristics, which was detectable by the image processing technique [18]. According to the studies above, the image analysis technique seems to be an efficient method for evaluating crumb structure, particularly the porosity of leavened bakery products.

From the above literature, it can be concluded that image processing is a well-established approach for bread porosity studies. However, the potential of this method has not yet been evaluated for many other leavened bakery products such as *pogácsa*, which has a softcore and crisp surface, unlike bread. Therefore, it can be an important topic of research to explore and/or standardize the efficacy level of this technique in assessing the porosity measurement of a unique-texture product such as *pogácsa*. Hence, this research work was designed to investigate the effect of formulations (using cheeses with different moisture content (MC)) and baking temperature (200 and 215 °C) on the porous structure and sensory properties of *pogácsa*, with the help of image analysis technique.

## 2. Materials and Methods

### 2.1. Materials

Margarine (Rama Co., Katowice, Poland), wheat flour (type BL55, Nagyititka Co., Budapest, Hungary), cottage cheese (Alfoldi-tej Co., Budapest, Hungary; Real Nature Co., Budapest, Hungary), salt, and yeast (Dr. August Oetker Co., Bielefeld, Germany) were procured from a local market.

### 2.2. Methods

#### 2.2.1. Sample Preparation

In order to prepare the *pogácsa* samples, margarine (900 g), cottage cheese (1000 g), salt (60 g), and yeast (60 g) were mixed using a laboratory blender (CPM 80–120, CP bakery equipment Co., Brescia, Italy). Cottage cheese was not added for preparing sample groups without the cheese. The next step was adding the wheat flour (1000 g) and kneading thoroughly until a homogenous soft dough was formed. The dough was allowed to rest overnight at 4 ± 1 °C. Afterward, the dough was rolled out to 6 mm in height and carefully plated into a flat shape to avoid margarine softening. A round-shaped mold (with 40 mm diameter) was used to cut the plated dough into round pieces. The molded samples were placed on baking trays and proofed for 20 min at 40 °C in a proofing chamber. After proofing, the samples were baked in the oven. The baking temperature was adjusted to 200 or 215 °C. These temperatures were selected based on our prior study [2].

To evaluate the effect of the addition of cheese on the quality of *pogácsa*, two kinds of cottage cheese with two different moisture content (MC) of 65% and 58% were used in dough formulations. One group of *pogácsa* was also prepared without cottage cheese. Totally, six different *pogácsa* groups (A1-3 and B1-3) were prepared as introduced in Table 1. *Pogácsa* samples were then cooled to room temperature, sealed in airtight plastic bags, and labeled with three-digit codes for further evaluation. The baking process was replicated three times, and six *pogácsa* samples were selected per blend. Therefore, eighteen samples were selected for physical, textural, and sensory evaluations of each *pogácsa* group.

#### 2.2.2. Physical Characteristics

*Pogácsa* mass was recorded by a digital balance (with 0.001 g accuracy). The volume was determined following the rapeseed displacement method (AACC, 2000). Density was calculated by dividing mass by volume. Specific volume was calculated from leaf volume (*V_L_*) and mass (*m_L_*), in agreement with the literature [19] (Equation (1)):

(1) $$\mathrm{Specific}\;\mathrm{volume}\;\left(\frac{{\mathrm{cm}}^{3}}{g}\right)=\frac{V_{L}}{m_{L}\;}$$

To determine the crust surface and crumb color of *pogácsa*, a chromameter (Minolta CR-310, Apeldoorn, The Netherlands) was used to measure L*, a*, and b* colorimetric parameters. For this purpose, the colorimeter was first calibrated by a standard white tile. *Pogácsa* samples were then cut into uniform pieces (3 cm diameter). Samples were placed in a circular template, and CIE L*a*b* was calculated by the device [20]. Measurements were performed at room temperature.

The moisture content of *pogácsa* samples was estimated by oven drying at 105 °C until constant weight [21].

The porosity of *pogácsa* samples was measured using the reference method designed for bread as described by Codex Alimentarius [22]. For this purpose, a regression model was built based on the table of MC and absolute density (*AD*) of bread to predict the *AD* of *pogácsa* for different MC. An empirical equation (Equation (2)) was selected to calculate absolute density, according to the value of the determination coefficient (*R*^2^ = 0.990).

(2) $$AD=\left(7.937\times 10^{-5}{\;\mathrm{MC}}^{2}\right)-0.012\;\mathrm{MC}+1.637$$

Porosity was estimated (Equation (3)) using initial mass (*M*_0_), predicted absolute density (*AD*), and sample volume (*V*).

(3) $$Porosity\;\left(\%\right)=1-\left(\frac{M_{0}}{AD\times V}\right)\times 100$$

#### 2.2.3. Image Processing

##### Image Acquisition

*Pogácsa* products were subjected to digital image analysis, according to the methodology described by Amani et al [2]. *Pogácsa* were cross-sectionally cut into two pieces. Image capturing of inside the *pogácsa* was carried out using an industrial camera (DFK 33UX273, The Imaging Source Co., Bremen, Germany). The camera was equipped with an F/1.8 lens (VS-2518VM) and a CMOS sensor. A fixed distance (30 cm) between the sample and the camera lens was used during image acquisition. Samples were illuminated using two white D65 type lamps on both sides of the imaging chamber. The angle between lamps and lens axis was approximately 45°. Images were captured on a white background using IC Capture programs (2.4, The Imaging Source Co., Bremen, Germany) and saved in lossless bitmap (BMP) format for subsequent image processing analyses.

##### Pore Characteristics Evaluation

**Figure 1:**
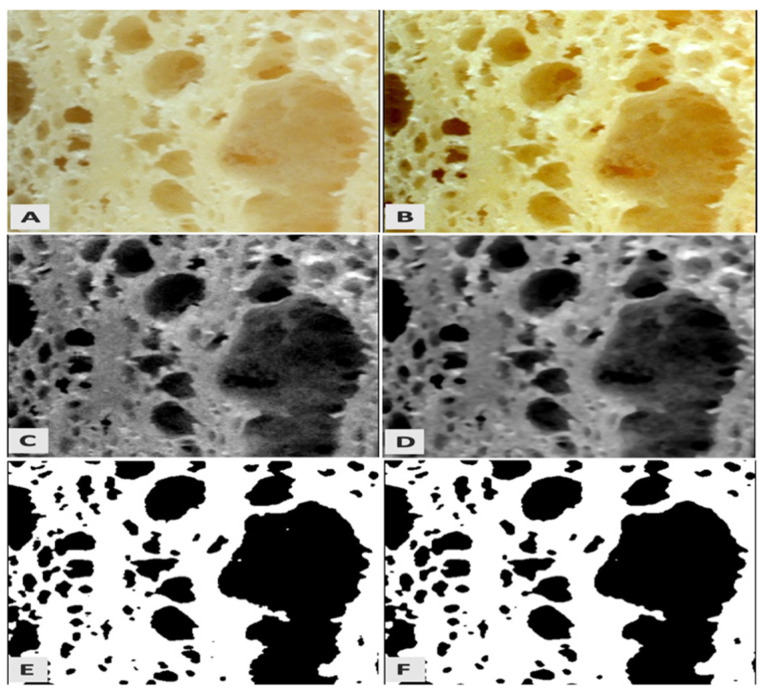
Image processing steps: ROI selection (**A**); contrast enhancement (**B**); desaturation (**C**); noise removal (**D**); segmentation (**E**); and noise removal (**F**).

To study the internal structure and pore characteristics of *pogácsa* samples, Fiji Image J software 1.44 (National Institutes of Health, Bethesda, MD, USA) was used for image analysis described by Schindelin et al. [23]. The optical resolution of the acquired images was 0.05 mm/pixel. The same area’s region of interest (ROI) was segmented from the background and cropped (Figure 1A). The Brightness/Contrast Image J command enhanced image contrast to improve the cropped image quality (Figure 1B). Afterward, the desaturation of images was done using the Intermodes filter (Figure 1C). As images captured by digital cameras contain numerous noises, a median filter was additionally used to filter out these noises (Figure 1D). After eliminating noises, the Otsu threshold was applied to pre-processed images (Figure 1E). The median filter was again applied to remove possible noises (Figure 1F). Finally, porosity characteristics were extracted from binary images, including the area of pores (mm^2^), pore volumetric ratio (PVR), the number of pores, and pore volume (mm^3^). In this method, noises of the original image might lead to creating additional holes in the processed image and erroneously consider the pore structure. However, excessive elimination of the noises can significantly reduce those errors.

##### 2.2.4. Mechanical Texture Analysis

The mechanical properties of *pogácsa* samples were determined via a TA-XT2i texture analyzer (Stable Microsystems, Godalming, UK). The texture analyzer was equipped with a 25 mm diameter cylindrical probe. The crust of the *pogácsa* sample was removed, and the remained part (4 cm diameter × 2 cm height) was axially placed on the platform. The instrument was adjusted to 0.5 mm/s test speed, 10 mm/s pre-test speed, 0.5 mm/s post-test speed, auto trigger with 2 g value. Exponent software (6.1.16.0, Stable Microsystems, Godalming, UK) was used for analyzing the data and the following parameters were calculated: cohesiveness (divided the area of force during the second and the first compression), hardness (the highest force of the initial compression), gumminess (multiplication of hardness by cohesiveness), resilience (divided the first compression upstroke area by its downstroke area), springiness (the ratio of the distance of maximum forces during the second and first compression), and chewiness (multiplication of gumminess and springiness) [24]. Ten test replicates were considered per each *pogácsa* group.

##### 2.2.5. Sensory Profiling

Sensory evaluation was carried out on *pogácsa* samples by 15 trained assessors (7 female, 8 male; aged between 22 and 45 years) at the Hungarian University of Agriculture and Life Sciences (Budapest, Hungary). The evaluation was performed under similar illumination conditions at room temperature according to ISO 8589 [25]. Samples were prepared for panelists and asked to complete questionnaires by scoring on sensorial attributes (including color, aroma/odor, taste, oiliness, chewiness, elasticity, hardness, crumbness, and pores structure). Samples were evaluated using a 5-point scale, “Just About Right” (JAR), where categories 1, 2 belong to “not enough” levels, 3 = JAR, and 4, 5 expresses “too much” levels. Assessors were also asked to evaluate the overall liking on a 9-point hedonic scale, where one presents” extremely dislike” and nine expresses “extremely like” of the *pogácsa*. Penalty analysis (PA) was used to analyze the JAR data, statically and mean drops (penalties) were calculated as the differences between means of the JAR and the mean of two categories of non-JAR (groups of “too much” and “not enough”), and consequently, to characterize the sensory attributes which cause variations in *pogácsa* [26,27].

##### 2.2.6. Data and Statistical Analyses

All statistical analyses were performed using IBM SPSS™ software (version 16.0, IBM Inc., Chicago, IL, USA). The Analysis of variance (ANOVA) was applied to the obtained data, and Duncan’s multiple range test was performed to detect the differences among means at the significance level of *p* < 0.05. The correlation coefficient (*R*) between visual and mechanical parameters was obtained using the Pearson correlation test. In order to investigate the relationship between MC and the porosity of the product, regression analysis was run, and the best fit was selected based on the *R*^2^ value. The penalty analysis of sensory data was carried out using XLSTAT software 2020.5 (Addinsoft, Paris, France).

## 3. Results and Discussion

### 3.1. Pore Characteristics

**Figure 2:**
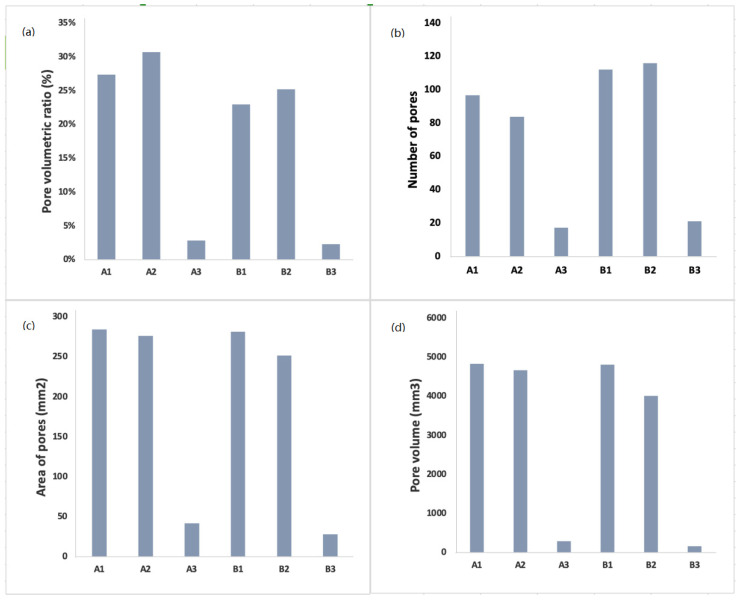
Pore characteristics of baked products formulated with different cottage cheese, (**a**) pore volumetric ratio (%); (**b**) number of pores; (**c**) the area of pores (mm^2^); (**d**) pores volume (mm^3^).

To study the effect of cottage cheese addition, with different moisture content and baking temperature on the internal structure of the *pogácsa*, pore characteristics including PVR, number of pores, area, and volume were analyzed. Since the crumb microstructure is a heterogeneous complex structure, it has a significant effect on the mechanical and sensorial properties of the final product. Pores with a well-developed structure could retain more leavened gas, resulting in a product with larger volume and lower crumb hardness. Therefore, pore’s structure evaluation of *pogácsa* can significantly affect the quality of the final product. As depicted in Figure 2a, sample A2 exhibited the highest PVR value (30.76%), followed by samples A1, B1, and B2 with PVR values of 27.43%, 25.21%, 22.97%. Samples A3 (2.84%) and B3 (2.30%) had the lowest PVR. Therefore, it can be assumed that using lower baking temperature (200 °C) and cheese with high MC (65%) during processing may substantially increase PVR values. Figure 2b shows the average number of pores in images of cross-sectioned samples. Sample B2 was found to have the highest number of pores with the value of 115, followed by samples B1 (112), A1 (96), and A2 (83). The lowest pores were observed for samples A3 (17) and B3 (21), which contained no cheese in their formulations. Variation in the values of PVR and the number of pores can be ascribed to the initial moisture level of the sample. The highest numbers of these two parameters were found in *pogácsa* samples with high moist cheese (65% MC). In addition, it can be assumed that an increase in baking temperature from 200 to 215 °C might cause an increase in the number of pores. The area of pores and the pores volume of *pogácsa* is presented in Figure 2c,d. Sample A1 had the highest values for both pores area (284.81 mm^2^) and pores volume (4835.17 mm^3^), whereas the lowest area (28.33 mm^2^) and volume of pores (160.53 mm^3^) were recorded for sample B3. The values obtained for the pore area indicate that using cheese in *pogácsa* formulation could generate bigger particle sizes occupied in the crumb of baked samples. For cheese-free samples, smaller areas might indicate a heterogeneous model of fragmentation [28,29]. Higher pore characteristics values, including PVR, number of pores, area, and volume of pores, have a positive effect on increasing the acceptability of *pogácsa* cake. The above observations showed that the lowest values for pore characteristics were achieved with cheese-free samples (A3 and B3), which was accounted in sensory tests as undesirable.

### 3.2. Physical and Textural Properties

Table 2 shows the results of textural properties of baked *pogácsa*. Significant variations (*p* < 0.05) in the chewiness, hardness, cohesiveness, gumminess, and springiness were observed among different groups. Hardness and chewiness values showed that higher baking temperature (215 °C) along with less moist cottage cheese (58% MC) in the formulation led to a harder crumb and higher chewiness compared to the samples baked at a lower temperature (200 °C). In the case of *pogácsa* groups prepared without cottage cheese (samples A3 and B3), sample B3, which was baked under higher baking temperature, had higher values for hardness and chewiness compared to sample A3 with lower baking temperature. This implies that the baking temperature could influence hardness and chewiness. Significant differences (*p* < 0.05) were also found among the cohesiveness values. Sample A1 had the highest cohesion value (0.61), while sample B3 had the lowest (0.32). In the case of gumminess, sample B1 showed a value of 694.81, which was noticeably higher than other groups, while the lowest was belonged to sample A3 (191.51). Changes in the springiness values could be related to the variation in hardness in such a way that increasing the hardness could cause a reduction in springiness and vice versa. This signifies that the increasing baking temperature (from 200 to 215 °C) might increase crumb hardness. Shittu et al. [30] found that baking conditions of time and temperature had a substantial impact on the hardness of bread crumbs. Their finding revealed that baking time was the most crucial element in increasing the bread crumb hardness [30].

As demonstrated in Table 2, the crumb moisture content was remarkably affected after using different recipes. High moisture cheese maximized the crumb moisture in *pogácsa* samples, whereas its absence minimized it. It must be considered that baking temperature affects the MC of the crumb [31].

Volume and density values also varied significantly (*p* < 0.05). However, no noticeable change was found in the volume of samples after changing the baking temperature. The main reason for this alteration could be related to the type of formulation used. For instance, samples without cheese (A3 and B3) showed the lowest volume values. While, after incorporation of cheese in formulation, samples A1 and B1, which were prepared with less moist cheese (58%) in the formulation, showed a higher volume than the groups A2 and B2 with high moist cheese (65%) in the recipe. Samples with minimum MC (samples A3 and B3) achieved the highest density. The low MC recorded for the samples above is due to the absence of cottage cheese in their recipe.

Changes in porosity due to an increase in baking temperature and the addition of cheese are shown in Table 2. The highest porosity value was found for sample B1 (72.75%), followed by samples A1 (69.63%), B2 (62.20%), A2 (53.36%), and B3 (38.83%). At the same time, the lowest value (32.66%) for this parameter was observed in sample A3, which was cheese-free and baked at 200 °C. Therefore, it can be interpreted that higher baking temperature had a significant effect as a result of increasing porosity in *pogácsa* cake. To our knowledge, only a few studies focused on assessing the impact of moisture content on porosity value. Esteller et al. [2] studied the effect of kefir addition on the porous quality of bread with image processing technique. They found that by decreasing the MC of the product, the porosity would increase [2]. Given the above explanations, higher porosity can be assumed to be associated with lower moisture content baked *pogácsa*.

Regarding the PVR factor, sample A2 exhibited the maximum value (30.76%), whereas the minimum belonged to samples A3 (2.84%) and B3 (2.30%). Significant differences were found among the sample groups prepared with cheese (A1, A2, B1, and B2) and cheese-free samples (A3 and B3). Therefore, it can be concluded that PVR can also increase together with increasing MC in *pogácsa*.

Color is also an important factor in increasing the overall acceptability of bakery products such as *pogácsa* [32]. This parameter can be affected by formulation and processing conditions. Factors such as dough characteristics (e.g., pH and water content) and baking conditions (e.g., temperature and time) might alter the product color. The standard CIE L*a*b* color space, a 3-dimensional spherical system, is typically used to describe the surface color of food materials [33]. The results of the color measurements of *pogácsa* are presented in Table 3. According to these data, L* values of crumb and crust decreased with increasing MC of the product. This indicated the impact of moisture content on the surface color. Cottage cheese contains protein which might contribute to a lower L* values in both crust and crumb. In addition, increasing the baking temperature could reduce crust L* values. The highest yellowness (b*) value was observed in sample A1 with values of 52.03 for the crust and 29.38 for the crumb, while the minimum value of b* was found for the crust (40.90) and crumb (20.71) of the sample A3. A significant difference was observed in a* values of crumb and crust of *pogácsa*. This implies that water migration from crumb to crust might increase a* values and thus change the visual appearance of the *pogácsa.* Hence, a* values of both crumb and crust were higher in groups B1, B2, and B3 compared to groups A1, A2, and A3. In align with our results, Shittu et al. [30] observed that color parameters such as L* of the bread crust significantly increased from31 to 72, by increasing both baking time and temperature from 20 to 40 min and 190 to 240 °C, respectively [30]. Overall, the above L*a*b* results suggested that baking temperature and formulation changes could cause variation in lightness, redness, and yellowness of *pogácsa*.

As shown in Table 4, significant correlation coefficients were observed among the visual parameter (PVR), MC, and instrumental textural features. The PVR and MC showed a significant negative correlation (*p* < 0.01) with gumminess, hardness, and chewiness, significant positive correlation (*p* < 0.01) with resilience, cohesiveness, and springiness. In addition, a strong positive correlation (*p* < 0.01) was observed between PVR and porosity results. It can be interpreted that changes in the image-based features, particularly PVR, are linked to the hardness variation of the *pogácsa* sample. In other words, the less firm the *pogácsa* texture, the higher PVR can get, and vice versa. In addition, the firmer crumb of the *pogácsa* is linked with lower MC. In line with the present study results, Morreale et al. [34] found a significant correlation between moisture content and mechanical texture parameters in gluten-free bread. They found a strong link between water content and crumb hardness [34]. In another study, Esteller et al. [3] examined the effect of Kefir concentration and proofing time on the pore’s quality of bread. They reported a significant correlation between physical properties and microstructure observed by image processing. In addition, they found a strong correlation between the microstructure of pores, hardness, and brightness of the white bread samples [3].

### 3.3. Sensory Evaluation

**Figure 3:**
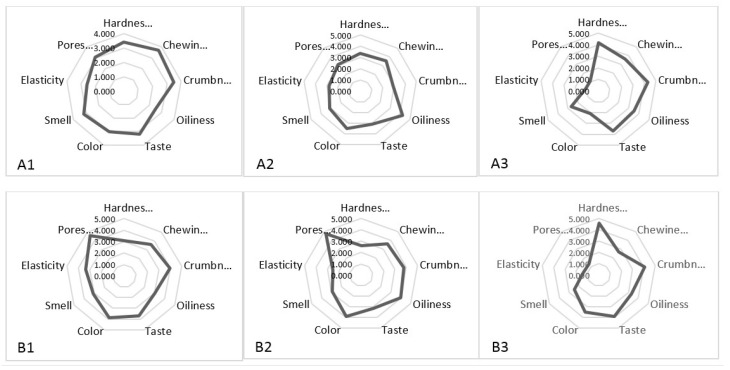
Average sensory profiles of *pogácsa* cake samples: (**A1**) baked at 200 °C, cheese with less MC (58%) in formulations; (**A2**) baked at 200 °C, cheese with high MC (65%) in formulations; (**A3**) baked at 200 °C, no cheese in formulation; (**B1**) baked at 215 °C, cheese with less MC (58%) in formulations; (**B2**) baked at 215 °C, cheese with high MC (65%) in formulations; (**B3**) baked at 215 °C, no cheese in formulation.

Results obtained from the sensory evaluation are illustrated in Figure 3. Significant differences were observed in nine parameters (taste, color, aroma/odor, oiliness, chewiness, hardness, elasticity, pores structure, and crumbness) among different *pogácsa* groups. Samples prepared without cheese (A3 and B3) had noticeably higher hardness. The oiliness in samples A2 and B2, formulated with higher moist cheese, was higher than in other groups. Among all *pogácsa* groups, B1 and B2 exhibited the highest value of pore structure. This might be due to increasing baking temperature (from 200 to 215 °C) on the crumb structure. Using higher temperatures for baking might lead to increased yeast activity, thereby generating a higher amount of CO_2_ in the processed dough. Similarly, a recent study by Amani et al. [2] confirmed the contributing role of baking temperature in increasing pore ratio in the *pogácsa* crumb. Those results indicated that increasing baking temperature from 200 to 230 °C could markedly increase the pore ratio from 0.45 to 0.56 [2].

**Figure 4:**
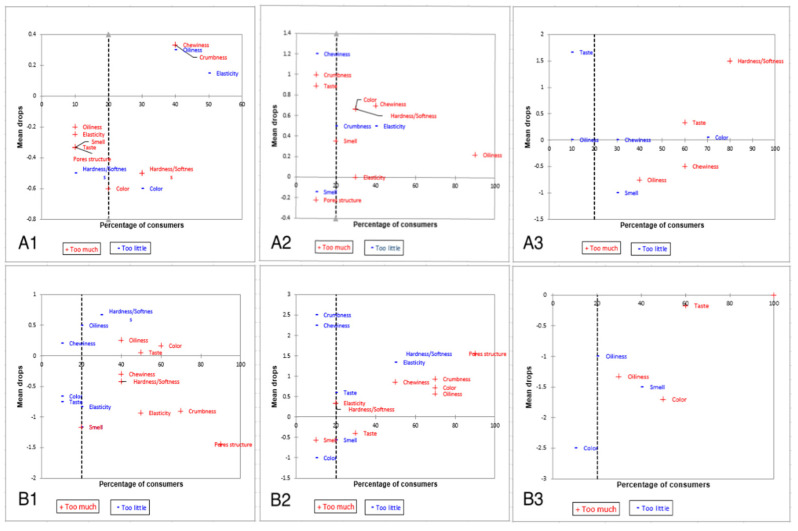
Mean drop charts of *pogácsa* samples (capital letters indicate the sample group). Red color corresponds to “too much”, while blue corresponds to “not enough” endpoints of the JAR scale. The dashed line corresponds to 20% of the consumers: (**A1**) baked at 200 °C, cheese with less MC (58%) in formulations; (**A2**) baked at 200 °C, cheese with high MC (65%) in formulations; (**A3**) baked at 200 °C, no cheese in formulation; (**B1**) baked at 215 °C, cheese with less MC (58%) in formulations; (**B2**) baked at 215 °C, cheese with high MC (65%) in formulations; (**B3**) baked at 215 °C, no cheese in formulation.

Penalty analysis (PA) was also performed to indicate the non-JAR attributes in different groups of *pogácsa*. The results of PA are shown in Figure 4. The plot illustrates the mean drops (variation in the average overall liking of optimal and non-optimal data of “too much” or “not enough” values) against the percentage of consumers. The top right subspace of the PA plot, which contains more than 20% of the customers, is the most important section. Other parts can be neglected due to not being significant [35]. In sample A1, 40–50% of panelists expressed elasticity and oiliness attributes as “not enough” and chewiness and crumbness as “too much”. A high percentage of consumers (80–100%) felt that the oiliness was “too much” in sample A2, which was prepared with higher moist cheese. In addition, 40% of respondents rated color, chewiness, and hardness as “too much” and elasticity as “not enough”. The hardness and taste were found to be “too much” by more than half of consumers in sample A3. The oiliness, taste, and color were considered as “too much” by 40–60% of panelists for the sample B1 and 20–30% of those felt that the hardness was “not enough”. More than half of the panelists in sample B2 found that the *pogácsa* sample was “not enough” hard and elastic. The values of “mean drops” in sample B3 were negative (below zero). This indicated that sample B3 was considered by panelists as sub-optimal compared to other *pogácsa* samples. Therefore, increasing the baking temperature from 200 to 215 °C along with removing the cottage cheese from formulation might lead to a marked decrease in the overall acceptability of baked *pogácsa*. Moreover, the higher baking temperature would probably result in an undesirable color score.

## 4. Conclusions

In the present study, the effects of baking temperature (200 and 215 °C) and addition of cottage cheese on *pogácsa* characteristics (porosity, image-based pore volumetric ratio, mechanical texture parameters, color, and sensorial attributes) were investigated. *Pogácsa* baked with higher temperature (215 °C) obtained higher porosity and color sensory score, higher porosity was measured by reference method and was firmer than *pogácsa* baked at a lower temperature (200 °C). Cottage cheese variation also affected the textural, physical, and sensorial properties of the samples. *Pogácsa* samples prepared using lower moist cheese (58%) showed the lowest density and crumb moisture values and the highest values for volume. Cheese-free samples seemed to have the lowest acceptability among panelists. A significant correlation (*p* < 0.01) was observed among the instrumental textural features, image-based parameter (PVR), and moisture content. The present study suggests that varying dough ingredients and baking temperature could result in significant differences in the textural and physical properties and generally in the pores’ crumb structure of *pogácsa*. Therefore, certain modifications of those factors, such as baking temperature and modifying the moisture content of cheese, can affect pores’ crumb structure. Image processing technique proved its ability to detect textural changes, which can provide an additional rapid and low-cost method in quality assessment. The presented approach can be utilized as a non-destructive technique combined with CT, MRI, or X-ray devices. However, there are some limitations/challenges such as (a) standardization of each sample based on its optimized processing conditions, (b) lack of portability of imaging system, (c) requiring software programming/adjustment according to physical nature and internal structural properties of the target product, and therefore, more systematic studies should be carried out to mitigate these limitations, thereby expanding the utilization of this technique in the bakery industry.

## Figures and Tables

**Table 1:** *Pogácsa* groups according to the formulation and baking temperature.

Sample Name	Baking Temperature (°C)	Cheese (% of Dough)	Cheese MC (%)
A1	200	33	58
A2	200	33	65
A3	200	-	-
B1	215	33	58
B2	215	33	65
B3	215	-	-

**Table 2:** Textural and visual properties, physical characteristics of *pogácsa* cake samples.

Parameters	A1	A2	A3	B1	B2	B3
Hardness (g)	571.99 ± 71.19 ^a^	471.67 ± 43.24 ^a^	5042 ± 338.91 ^c^	1441.1 ± 131.65 ^b^	674.46 ± 57.84 ^a^	7778.8 ± 851.60 ^d^
Cohesiveness	0.61 ± 0.02 ^e^	0.54 ± 0.01 ^d^	0.40 ± 0.03 ^b^	0.49 ± 0.03 ^c^	0.53 ± 0.01 ^d^	0.32 ± 0.02 ^a^
Gumminess	371.43 ± 31.86 ^c^	259.29 ± 23.70 ^b^	191.51 ± 11.11 ^a^	694.81 ± 68.20 ^d^	359.69 ± 30.10 ^c^	256.7 ± 20.07 ^b^
Chewiness	309.71 ± 33.79 ^a^	232.98 ± 26.78 ^a^	1256.08 ± 36.69 ^c^	568.98 ± 91.78 ^b^	311.83 ± 35.57 ^a^	1526.62 ± 220.53 ^d^
Springiness (%)	86.30 ± 3.05 ^cd^	89.94 ± 7.83 ^d^	62.74 ± 3.58 ^b^	83.61 ± 8.75 ^c^	85.97 ± 3.13 ^cd^	55.72 ± 1.83 ^a^
Crumb MC (%)	36.09 ± 3.40 ^b^	47.18 ± 4.96 ^c^	15.37 ± 0.61 ^a^	35.41 ± 0.97 ^b^	43.66 ± 5.28 ^c^	14.81 ± 0.37 ^a^
Volume (cm^3^/g)	63.00 ± 4.58 ^d^	49.00 ± 1.00 ^c^	29.00 ± 2.00 ^b^	61.00 ± 1.00 ^d^	48.66 ± 2.08 ^c^	24.33 ± 1.15 ^a^
Density	0.24 ± 0.02 ^a^	0.29 ± 0.01 ^b^	0.46 ± 0.00 ^c^	0.23 ± 0.01 ^a^	0.27 ± 0.01 ^ab^	0.62 ± 0.05 ^d^
Porosity (%)	69.63 ± 1.58 ^e^	53.36 ± 5.15 ^c^	32.66 ± 3.34 ^a^	72.75 ± 1.48 ^f^	62.20 ± 1.49 ^d^	38.83 ± 6.32 ^b^
PVR (%)	27.43 ± 5.09 ^c^	30.76 ± 7.67 ^d^	2.84 ± 0.68 ^a^	22.97 ± 3.91 ^b^	25.21 ± 1.65 ^bc^	2.30 ± 0.53 ^a^

Different superscript letters in each row indicate significant differences (*p* < 0.05).

**Table 3:** Colorimetric parameters of the *pogácsa* cakes.

Sample	L*		a*		b*	
	Crumb	Crust	Crumb	Crust	Crumb	Crust
A1	92.10 ± 1.63 ^c^	89.80 ± 3.29 ^c^	−0.95 ± 0.49 ^b^	9.09 ± 1.10 ^a^	29.38 ± 1.23 ^d^	52.03 ± 0.54 ^c^
A2	85.16 ± 1.34 ^b^	85.98 ± 1.42 ^b^	−0.16 ± 0.01 ^c^	11.92 ± 0.42 ^bc^	26.78 ± 2.01 ^c^	51.83 ± 2.16 ^c^
A3	83.54 ± 0.49 ^ab^	71.93 ± 0.15 ^a^	−0.15 ± 0.04 ^c^	9.94 ± 0.27 ^ab^	20.71 ± 0.97 ^a^	40.90 ± 1.15 ^a^
B1	85.36 ± 1.94 ^b^	72.92 ± 0.66 ^a^	−1.39 ± 0.08 ^a^	27.45 ± 2.47 ^e^	31.90 ± 1.92 ^d^	49.99 ± 1.94 ^c^
B2	84.23 ± 0.60 ^ab^	70.47 ± 2.54 ^a^	−1.12 ± 0.06 ^ab^	23.61 ± 0.64 ^d^	30.83 ± 1.09 ^d^	44.87 ± 1.66 ^b^
B3	82.22 ± 1.66 ^a^	69.71 ± 2.75 ^a^	0.53 ± 0.12 ^d^	13.20 ± 0.57 ^c^	24.05 ± 0.56 ^b^	42.37 ± 1.15 ^ab^

Data in columns with different superscript letters are significantly different (*p* < 0.05).

**Table 4:** Correlation coefficients of volumetric pore ratio (PVR) and moisture content (MC) with instrumental texture (TPA) parameters and porosity of *pogácsa* cakes.

Parameters	Hardness (g)	Resilience (%)	Cohesiveness	Springiness (%)	Gumminess	Chewiness	Porosity
PVR	−0.90 **	0.87 **	0.84 **	0.87 **	−0.92 **	−0.92 **	0.71 **
MC	−0.89 **	0.83 **	0.82 **	0.90 **	−0.92 **	−0.92 **	-

** Correlation is significant at *p* < 0.01.

## Data Availability

The data generated from the study is clearly presented and discussed in the manuscript.
